# Malignant Eccrine Spiradenoma of the External Auditory Canal: A Case Report and Literature Review

**DOI:** 10.3389/fonc.2020.574112

**Published:** 2021-01-05

**Authors:** Dong You, Zhao Ma, Jing Liu, Xiao Song, Wei Dong

**Affiliations:** ^1^ Department of Radiation Oncology, Yantai Yuhuangding Hospital, Yantai, China; ^2^ Department of Pathology, Yantai Yuhuangding Hospital, Yantai, China; ^3^ Department of Pathology, Rongcheng City People’s Hospital, Rongcheng, China

**Keywords:** malignant eccrine spiradenoma, external auditory canal, sweat gland tumor, case report, review

## Abstract

Spiradenocarcinoma, or malignant eccrine spiradenoma (MES), is a rare sweat gland tumor originating from eccrine sweat glands. To the best of our knowledge, only two cases of MES of the external auditory canal have been reported to date. Here, we report a third case of MES located in the external auditory canal.

## Introduction

Spiradenocarcinoma (SA), or malignant eccrine spiradenoma (MES), is a rare sweat gland tumor originating from eccrine sweat glands that was first reported by Dabska (1) in 1972. MES is usually found on the trunk, extremities (2), or head and neck region (3), and it can develop *de novo* (3, 4) or arise from an eccrine spiradenoma (ES) (2, 5–8). The incidence of SA is similar in both sexes, and ES is more common in elderly individuals, presenting in patients with a mean age of 59 years (9). MES is regarded as a rare, very aggressive tumor with a poor prognosis because it is prone to relapse and metastasize to the lymph nodes, bones, lungs, and brain. To the best of our knowledge, only three cases of MES of the ear have been reported to date, including two cases located in the external auditory canal. We encountered a patient with an MES in the external auditory canal, a rare site according to previous reports. The etiology of such tumors is unknown, and a treatment strategy has not yet been established. Therefore, it is essential to accumulate case reports for the identification of mechanisms underlying the pathogenesis and disease progression of MES and to facilitate the diagnosis and development of an effective treatment.

## Case Description

**Figure 1:**
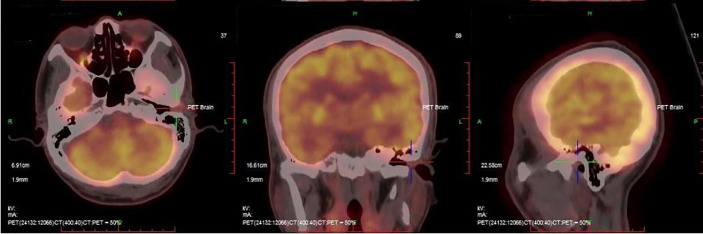
PET-CT scan of the whole body demonstrated the ﬂuorodeoxyglucose (FDG)-avid mass (maximum standard uptake volume (SUVmax 2.6).

The patient was a fifty-six-year-old man who initially complained of swelling in his left external ear canal. Over the previous year, he had gradually experienced severe left trigeminal neuralgia, which was occasionally accompanied by a sensation of ear fullness. On physical examination, the lesion was a solitary nodule, with a maximum diameter of 1.6 cm. The lesion was a tender, smooth, firm swelling nodule that was skin-colored with a wide base, and it had not ulcerated the left external auditory canal, which was covered with intact skin. The lesion occluded the ear canal and obstructed the view of the tympanic membrane. The patient’s hearing in this ear was slightly affected. There was no sign of regional or distant metastasis on examination before surgery. The remaining clinical imaging examinations, including head, neck and cranial nerve examinations, were normal. Routine laboratory test results, including those of liver function, kidney function, total electrolyte levels, coagulation function and complete blood counts, remained within normal limits. A high-resolution positron emission tomography (PET)-computed tomography (CT) scan of the whole body demonstrated the fluorodeoxyglucose (FDG)-avid mass described above (maximum standard uptake volume (SUVmax 2.6), while no involvement of the bony canal walls was identified (**Figure 1**). The patient had a history of being physically fit and did not have a personal or family history of skin tumors. Taken together, the results demonstrated that the patient had no evidence of local positive lymph nodes or distant metastases, and the lesion was limited to the external auditory canal. The patient underwent wide excision of the lesion with clear margins under general anesthesia. The postoperative course was normal and uneventful. The patient was followed up over a period of 6 months, and no evidence of local recurrence or systemic metastasis was clinically observed. The patient was instructed to report for follow-up after another 6 months to ensure that there is no early recurrence.

## Diagnostic Assessment

The surgical specimen was fixed in 10% formalin and embedded in conventional paraffin blocks. Tissue sections (2 µm thick) were obtained for standard immunohistochemical staining according to routine protocols. The sections were placed in absolute ethanol, followed by 95% ethanol for 2 min, 80% ethanol for 2 min, and distilled water for 5 min; then, the sections were rehydrated in distilled H_2_O_2_ after exposure to a graded ethanol sequence. Antigen retrieval was used to enhance CK-7, EMA, P63, Vimentin, ER, PR, Her-2, S-100, CD56, NSE, Syn, CgA and Ki-67 immunohistochemistry by placing the sections in citrate buffer (pH 6.0) under high pressure for 3 min. Then, the sections were washed and incubated for 1 h at room temperature. The sections were washed in phosphate-buffered saline and then incubated with secondary antibody for 20 min at room temperature. After washing, the sections were stained with DAB until the desired stain intensity developed; then, the sections were mounted before observation by light microscopy. Positive and negative controls were included for each immunohistochemical run.

**Figure 2:**
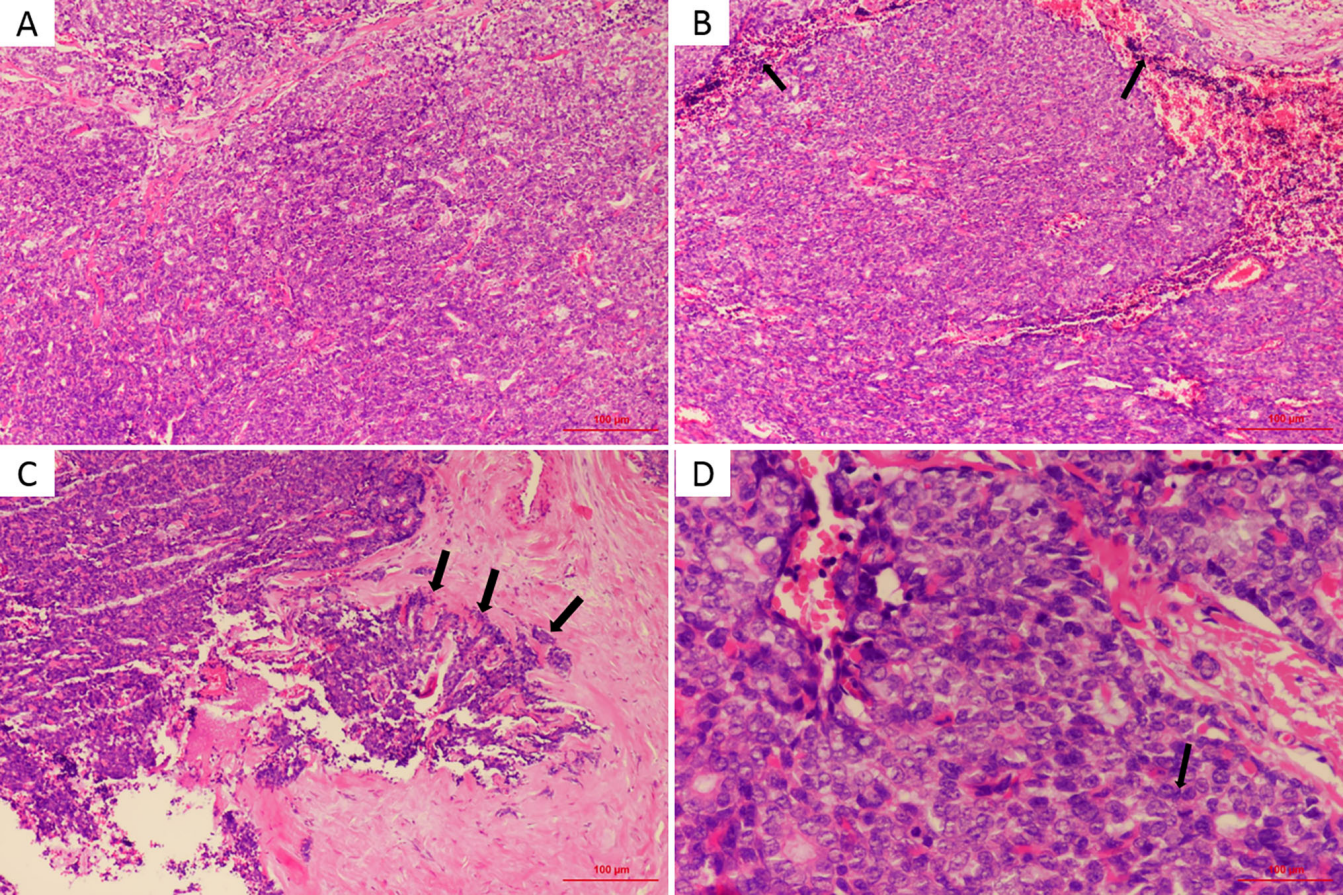
**(A)** Tumor mainly composed of pale and dark cells, which are similar to basaloid in benign part (HE, × 100). **(B)** Lymphocytes (arrows) were infiltrated around the central tumor area (HE, × 100). **(C)** Partial capsule is incomplete (arrows), forming a focal infiltration (HE, × 100). **(D)** The mitotic activity (arrow) was high with a mitotic index count estimated at 10∼15 mitosis per 10 high power field (HE, × 400).

Surgical pathologic findings led to the diagnosis of primary MES of the external auditory canal. Histological examination revealed that the tumor was located in the dermis and subcutis with no involvement of the epidermis and no residual tumor in the tumor-free margin. The benign area of tumor tissue showed typical ES histological characteristics, consisting of two types of cells: small cells with darkly staining nuclei surrounding larger cells with pale cytoplasm (**Figure 2A**). Most of the tumor stroma in the benign area was loose edematous tissue that was partially characterized by glassy lesions. Lymphocytes had infiltrated around the central tumor area (**Figure 2B**), and the capsule was incomplete, forming a focal invasion (**Figure 2C**) at the transition zone between the benign and malignant components under review. Cell division could easily be located in the malignant area. High mitotic activity was observed, with a mitotic index count estimated at 10–15 mitotic cells per 10 high-power fields (**Figure 2D**), and some of these cells were atypical. The malignant area exhibited a higher nuclear–cytoplasmic ratio, sometimes accompanied by focal tissue necrosis. There was no evidence of lymphovascular invasion. The pathologist did not find clear signs of vascular invasion. The tumor was positive for CK-7, EMA, P63, and Vimentin but negative for ER, PR, Her-2, S-100, CD56, NSE, Syn and CgA (details as shown in **Table 1**). The Ki-67 index was estimated at 30–50%. Taken together, the overall results indicated the features of ES with two strikingly different cell types, a loose and edematous tumor stroma and foci of glassy lesions. However, the presence of elevated mitotic counts, an incomplete capsule, and foci of tissue necrosis supported a malignant diagnosis. In conclusion, all findings reviewed favored a diagnosis of MES.

**Table 1:** Results of antibodies employed in the immunohistochemistry study.

Antibody	Clone	Dilution	Result	Bought from
Anti CK7	UMAB161	1:100	+	Beijing Zhongshang Jinqiao Biotechnology Co., Ltd
Anti EMA	UMAB57	1:100	+	Beijing Zhongshang Jinqiao Biotechnology Co., Ltd
Anti P63	UMAB4/4A4	1:100	+	Beijing Zhongshang Jinqiao Biotechnology Co., Ltd
Anti Vimentin	UMAB159	1:50	+	Beijing Zhongshang Jinqiao Biotechnology Co., Ltd
Anti ER	EP1	1:100	–	Beijing Zhongshang Jinqiao Biotechnology Co., Ltd
Anti PR	6F11	1:50	–	Beijing Zhongshang Jinqiao Biotechnology Co., Ltd
Anti Her-2	4B5	1:100	–	Roche
Anti S-100	Rabbit polyclonal	1:100	–	Beijing Zhongshang Jinqiao Biotechnology Co., Ltd
Anti CD56	UMAB83	1:100	–	Beijing Zhongshang Jinqiao Biotechnology Co., Ltd
Anti NSE	5E2	1:100	–	Beijing Zhongshang Jinqiao Biotechnology Co., Ltd
Anti Syn	EP158	1:100	–	Beijing Zhongshang Jinqiao Biotechnology Co., Ltd
Anti CgA	LK2H10	1:100	–	Beijing Zhongshang Jinqiao Biotechnology Co., Ltd
Anti Ki-67	UMAB107	1:100	30–50%	Beijing Zhongshang Jinqiao Biotechnology Co., Ltd

## Discussion

Here, we describe the third case of MES arising in the external auditory canal. ES was first described by Kersting and Helwig in 1956 as a benign neoplasm with a slow, indolent course (10). Compared to other benign sweat gland tumors, SA is localized at deeper sites, sometimes completely embedded in the subcutaneous adipose tissue, resulting in no clinically apparent significant change in the skin surface in most cases; therefore, SA might be mistaken for amyloidoma or a mesenchymal tumour. As late as 1972, Dabska (1) reported the first malignant transformation of ES (MES). In the majority of cases, the clinical manifestations of ES are similar to those of SA; it is usually a solitary, intradermal, firm, and often painful nodule, often appearing on the ventral aspect of the upper body. Moreover, several studies have reported multiple nodules (11) or a linear zosteriform pattern (12–14). The typical clinical manifestation of MES is malignant transformation from a long-standing benign ES lesion with recent rapid growth, color change, erythema, ulceration, new-onset pain and bleeding (1, 15–20). However, rare reports of a new MES without a previous stable lesion have also been published (3, 4, 21, 22). Here, we reported a *de novo* MES located in the external auditory canal. No relevant results were found in the patient’s routine health examination performed 1 year before the diagnosis. Whether it is *de novo* or arises from a benign ES, MES is associated with aggressive behavior, a high recurrence rate, and subsequent development of fatal metastases. Malignant transformation usually requires 20 to 30 years from the onset of the initial benign lesion, with an extremely variable range from 6 months to 70 years (23). There are two main forms of malignant transformation. One is the gradual transition of benign components to malignant components. The cell components transform from two benign cell types to single cancer cells with some common structural features of SA, including glassy lesions. The two typical cell types are replaced with cells that exhibit homing. Additionally, ductal structures and unclear boundaries are observed. The other type of malignant transformation exhibits malignant areas that are adjacent to benign regions without obvious transition zones. In our case, the capsule was incomplete, forming a focal infiltration, which indicates its malignancy, as shown in **Figure 2C**. The diverse histological findings usually suggest squamous cell carcinomas, seven-like disease and adenomatous ductal carcinomas. A diagnosis based on the above features is easily missed in the early stage or when the lesion is relatively limited. As a result of its rarity, the correct diagnosis of MES is challenging, particularly when it occurs in a rare location such as the external auditory canal. Histopathological diagnosis *via* an excisional biopsy or surgical resection specimen is the gold standard, and the presence of benign ES adjacent to malignant proliferation is the key basis for the diagnosis of MES. In most reported cases, the diagnosis was made based more on morphological appearance than on immunohistochemical evaluation. The expression of S-100, CEA, Ki-67 and other cancer-related proteins may provide some assistance in diagnosis, but their patterns of expression vary in different cases (24, 25) in the current literature. To date, only three cases of MES arising in the ear have been reported, including two located in the external auditory canal. The case presented here is the third case.

Imaging procedures, including CT scans, magnetic resonance imaging (MRI), PET-CT, chest X-ray, ultrasound, mammography and scintigraphy of the lymphatic drainage area, have been used to further define the size and extent of these primary neoplasms (26). In this case, we found a higher SUVmax (2.6) in the avid mass, while no involvement of the bony canal walls was identified (**Figure 1**), indicating that PET-CT is helpful in diagnosing and defining the scope of the tumour.

At present, there are no clear guidelines for the treatment of MES. A meta-analysis concluded that a tumor-free margin upon surgical excision is the definitive treatment for patients without lymph node metastasis, with 100% disease-free survival at a mean follow-up of 33 months (27). In addition, seven patients (six cases) with lymph node positivity but no distant metastasis treated with surgical and lymph node dissection remained disease-free at the final follow-up evaluation (mean, 47 months; standard deviation, 36 months; range, 2–97 months). Furthermore, in the other 24 cases with distant metastases, there were no significant differences in survival among patients who underwent local resection and surgery followed by adjuvant chemoradiotherapy. In another literature review, only distant metastasis indicated poor prognosis (16). Based on the current literature describing MES, wide surgical excision with 1-cm margins and depth down to the fascia or Mohs micrographic surgery (MMS) are the main treatment options (3, 28). Compared to surgical excision, MMS offers a better solution for cases that occur in cosmetically sensitive areas, decreasing the size of the final scar (29–32). Regional lymph node dissection has been advocated for clinical suspicion of positive regional lymph nodes on radiologic imaging before surgery (16, 27). Moreover, a meta-analysis by Andreoli and Itani (27) found that sentinel node biopsy may benefit patients with clinically absent lymph node involvement before treatment. Staiger et al. (26) reported that wide resection with tumor-free margins resulted in fewer recurrences and deaths due to unresected lesions (recurrence 23 *vs.* 43%, death 8 *vs.* 43%). Similarly, as early as 1997, Tay et al. (33) reported recurrence rates of up to 39% if MES was left untreated. Regional lymph nodes and the lungs, brain, and liver are the most common metastatic sites (4), while some studies have reported dissemination to the skin, spinal cord, and parotid gland (5, 22, 34). Insufficient data limit the formulation of a clinical approach after excision, including adjuvant radiotherapy and chemotherapy. Sweat gland tumours are generally considered radioresistant, implying the limited role of radiotherapy in the treatment of MES (33, 35, 36). Cabbabe (37) reported an eccrine carcinoma patient showing a good response to 7,400 rad, but Meyer et al. (22) attributed the unique success to its particular location, which allowed for the use of a massive radiation dose. Due to the rarity of MES, experience with chemotherapy is limited (15), and fluorouracil (5-Fu) (33), epirubicin (21), and ifosfamide (21) resulted in little curative effect without exception. Follow-up should be performed every 3 months following initial treatment (16, 26, 38). For cases with ER-positive status, tamoxifen therapy at a long-term maintenance dosage of 20 mg per day may provide a reasonable adjunctive therapeutic option to surgical treatment based on the literature, with one patient remaining disease-free at 41 months (39) and the second relapsing after 36 months (33). Another study also reported (16) that one patient treated with continuous tamoxifen therapy after tumor resection of an ER-positive MES localized in the upper arm and lymphadenectomy was free of recurrence after 41 months of follow-up.

In conclusion, we described here the third case of MES localized in the external auditory canal. The primary treatment of MES is wide local excision, and evidence of the benefits of adjuvant therapy, including radiotherapy, chemotherapy, and tamoxifen, is limited due to the rarity of MES. Hence, more information is needed about this rare tumor and its biological behavior to develop an optimal treatment modality in the future. We will continue to focus on the follow-up of this patient and report the progress of the case over time.

## Data Availability Statement

The original contributions presented in the study are included in the article/supplementary materials, further inquiries can be directed to the corresponding author.

## Ethics Statement

The studies involving human participants were reviewed and approved by the Medical Ethics Committees of Yantai Yuhuangding Hospital Affiliated to Qingdao University. The patients/participants provided their written informed consent to participate in this study.

## Author Contributions

DY collected the clinical data of the case and wrote the manuscript. ZM reviewed the related literature and helped in editing the manuscript. JL and XS performed the hematoxylin and eosin and immunohistochemistry procedures. WD edited and critically revised the manuscript for valuable intellectual content. All authors contributed to the article and approved the submitted version.

## Funding

This research was supported by the Yantai Science and Technology Bureau Support Grant/Science and Technology Innovation Development Project (2020MSGY085).

## Conflict of Interest

The authors declare that the research was conducted in the absence of any commercial or financial relationships that could be construed as a potential conflict of interest.

## Abbreviations

SA, spiradenocarcinoma; MES, malignant eccrine spiradenoma; ES, eccrine spiradenoma; CT, computed tomography; MRI, magnetic resonance imaging; PET-CT, positron emission tomography-CT; MMS, Mohs micrographic surgery; FDG, fluorodeoxyglucose; SUVmax, maximum standard uptake volume; 5-Fu, fluorouracil; ER, estrogen receptor.
